# Excellence in forensic psychiatry services: international survey of qualities and correlates

**DOI:** 10.1192/bjo.2023.578

**Published:** 2023-10-13

**Authors:** Patrick McLaughlin, Philip Brady, Felice Carabellese, Fulvio Carabellese, Lia Parente, Lisbeth Uhrskov Sorensen, Ingeborg Jeandarme, Petra Habets, Alexander I. F. Simpson, Mary Davoren, Harry G. Kennedy

**Affiliations:** National Forensic Mental Health Service, Central Mental Hospital, Portrane, Dublin, Ireland; and DUNDRUM Centre for Forensic Excellence, Academic Department of Psychiatry, Trinity College Dublin, Dublin, Ireland; Interdisciplinary Department of Medicine, Section of Criminology and Forensic Psychiatry, University of Bari ‘Aldo Moro’, Puglia, Italy; Department for Forensic Psychiatry, Aarhus University Hospital Psychiatry, Aarhus, Denmark; and Institute of Clinical Medicine, Health, Aarhus University, Aarhus, Denmark; Knowledge Centre for Forensic Psychiatric Care (KeFor), OPZC Rekem, Rekem, Belgium; and KU Leuven, Leuven, Belgium; Knowledge Centre for Forensic Psychiatric Care (KeFor), OPZC Rekem, Rekem, Belgium; and Tilburg University, Tilburg, The Netherlands; Centre for Addiction and Mental Health, Toronto, Ontario, Canada; and Department of Psychiatry, Temerty School of Medicine, University of Toronto, Toronto, Canada; National Forensic Mental Health Service, Central Mental Hospital, Portrane, Dublin, Ireland; DUNDRUM Centre for Forensic Excellence, Academic Department of Psychiatry, Trinity College Dublin, Dublin, Ireland; and Interdisciplinary Department of Medicine, Section of Criminology and Forensic Psychiatry, University of Bari ‘Aldo Moro’, Puglia, Italy; DUNDRUM Centre for Forensic Excellence, Academic Department of Psychiatry, Trinity College Dublin, Dublin, Ireland; Interdisciplinary Department of Medicine, Section of Criminology and Forensic Psychiatry, University of Bari ‘Aldo Moro’, Puglia, Italy; and Institute of Clinical Medicine, Health, Aarhus University, Aarhus, Denmark

**Keywords:** Excellence, quality, forensic, psychiatry, services

## Abstract

**Background:**

Excellence is that quality that drives continuously improving outcomes for patients. Excellence must be measurable. We set out to measure excellence in forensic mental health services according to four levels of organisation and complexity (basic, standard, progressive and excellent) across seven domains: values and rights; clinical organisation; consistency; timescale; specialisation; routine outcome measures; research and development.

**Aims:**

To validate the psychometric properties of a measurement scale to test which objective features of forensic services might relate to excellence: for example, university linkages, service size and integrated patient pathways across levels of therapeutic security.

**Method:**

A survey instrument was devised by a modified Delphi process. Forensic leads, either clinical or academic, in 48 forensic services across 5 jurisdictions completed the questionnaire.

**Results:**

Regression analysis found that the number of security levels, linked patient pathways, number of in-patient teams and joint university appointments predicted total excellence score.

**Conclusions:**

Larger services organised according to stratified therapeutic security and with strong university and research links scored higher on this measure of excellence. A weakness is that these were self-ratings. Reliability could be improved with peer review and with objective measures such as quality and quantity of research output. For the future, studies are needed of the determinants of other objective measures of better outcomes for patients, including shorter lengths of stay, reduced recidivism and readmission, and improved physical and mental health and quality of life.

Excellence is that quality that drives continuously improving outcomes for patients. To have a real meaning, excellence must be measurable. In forensic mental health services it could be measured in terms of four levels of organisation and complexity (basic, standard, progressive and excellent) across seven domains: (a) values and rights, (b) clinical organisation, (c) consistency, (d) timescale, (e) specialisation, (f) routine outcome measures and (g) research and development.^1^ These seven domains are derived from examples in other fields of medicine.^1–6^ In this study we intend to use an excellence scale based on achievable goals that generate measures and examine determinants of the scale score.

Forensic mental health services are high cost, low volume and high risk, and therefore intended to yield high value in health gains.^4,7^ These services treat people with severe life-shortening illnesses.^8,9^ A problem for forensic mental health services is that research leading to improved outcomes is difficult. Case mix is often heterogeneous for diagnosis, with extensive comorbidities,^10,11^ and heterogeneous for need for therapeutic security itself;^12–14^ evidence is lacking for what is effective treatment in several domains,^15–17^ and there is evidence of variable practice across similar services and strained accommodations to patient-centred approaches.^18–21^ There is evidence of progress in some areas.^22,23^ Organisations differ in their models of care^24^ and this may affect outcome, since the model of care is the framework that enables treatment delivery and ensures the measurement of outcomes.^4^ Process improvement has stagnated in forensic mental health services as elsewhere in psychiatry, with little attention to standards for treatment, whether treatment is delivered in sufficient quantity and whether treatment leads to measured improvement in real outcomes.^25^

To continuously improve ‘hard’ outcomes for patients, such as life expectancy and functional gains, a direct or indirect measure is required that monitors relevant standards. A starting point is the Organisation for Economic Co-operation and Development (OECD) definition of research and development:^26^ the activity must be novel, creative, uncertain, systematic, and transferable or reproducible or both. The OECD refers to three types of research and development activity: basic research, applied research and experimental development. We are concerned here with applied research and experimental development. In forensic psychiatry, applied research is directed towards a ‘hard’ outcome.^4,27^ Experimental development is directed towards producing or improving health gains for patients,^26^ either directly through better clinical practices^28^ or indirectly through better service processes.^29^

There are comprehensive accounts of specialist health services in tiers proportionate to the size of population served. These can be described also in referral pathways from primary care to general hospital services.^30,31^ Typically, to achieve critical mass, this organisation of services involves progressive centralisation, from district to regional to national or supra-national. A minimum level of activity is necessary to ensure continually refreshed clinical skills for clinicians expert in less common conditions or treatments, experience for trainees and specialist capacity for research and development.^5^

In a previous paper we proposed that forensic mental health services for a population can be described in terms of four levels of organisation and complexity.^1^ These levels are not the same as the tiers that describe a method of organising mental health services according to population size (Table 1). Level 1 (basic) corresponds to a system operated by individual practitioners or independent expert clinical groups. Level 2 (standard) describes local multidisciplinary teams (MDTs) or groups of services. Level 3 (progressive) refers to integrated community and hospital services with a broad service mandate. Level 4 (excellent) refers to academically led and productive centres of excellence. Seven domains characterising these levels were derived from an iterative process informed by formats or structures for models of care and process mapping. These domains are: (a) values and rights, (b) clinical organisation, (c) consistency, (d) timescale, (e) specialisation, (f) routine outcome measures and (g) research and development. Timescale here refers to individual case management and operational organisation of the service at day-to-day levels (for review and decision-making), weekly, monthly, quarterly and annually, and, at level 4, five-year plans and continuous cycles.

**Table 1:** Framework for characterising level of excellence of forensic mental health services

Domain	Level 1: basic	Level 2: standard	Level 3: progressive	Level 4: excellent
Values and rights	Individualisation	Professionalism	Consistency and evidence-based practice to address delivery	As level 3, plus academic resources and skills
Clinical organisation	Independent clinicians/disciplines	Multidisciplinary teams	Hospital governance	As level 3, plus national and international networks
Consistency	None	Within team only; some manualised treatment programmes	Admissions criteria and admission panels, evidence-based leave and tribunal reports	As level 3, plus increased measurement, stage of progression, neuropsychological, neuropharmacological and genetic profiling
Timescale	Day to day	Week to week	Monthly, quarterly, annual	Five-year plans and continuous cycles
Specialisation	Patient to patient Qualitative	Small units – gender, diagnostic, stratified therapeutic security levels	Medium-term intensive; longer-term slow stream; precision medicine	‘Treatment as usual’ is defined and disseminated; aspires to personalised medicine
Routine outcome measures	Qualitative	Dynamic only; risk, needs assessment	Functional outcomes linked to evidence-based governance reporting	As level 3, plus 6-monthly routine outcome measurement
Research and development	Case studies	Case series	Retrospective and prospective cohort studies	As level 3, plus multi-centre cohort studies and randomised controlled trials; population-based epidemiology; molecular, imaging and epidemiological translational research

Source: derived from Kennedy et al (2019).^1^

Other parts of the health sector have been more effective at implementing consistent service standards, including treatment as usual (TAU) linked to clinical trials and services aimed at driving improvement. The achievement of high quality in the provision of TAU is the primary goal of level 3 services. The advancement of new knowledge, interventions and services to improve outcomes is the mission of level 4 services.

This mapping study aims to fill the gaps in knowledge concerning forensic psychiatric research and development, translation, teaching and training,^15–17^ thus complementing quality studies.^32^ We remain focused on systematic ways of improving measurable real-world outcomes relevant to patients, such as survival and functional recovery, personal recovery, symptomatic recovery and forensic recovery.^4^

The aim of this study was to use a survey instrument with acceptable psychometric properties to examine the characteristics of forensic mental health services that related to excellence. We developed an excellence scale with psychometric properties that would allow forensic services to measure their current practices across the seven domains outlined above, and in future to assess progress. We tested which objective features of forensic services might relate to excellence: for example, university linkages, service size and integrated patient pathways across levels of therapeutic security.

## Method

A survey instrument was devised by a modified Delphi process.^33^ Forensic leads, either clinical or academic, in 48 forensic services across 5 jurisdictions completed the questionnaire.

### Ethics

The authors assert that all procedures contributing to this work comply with the ethical standards of the relevant national and institutional committees on human experimentation and with the Helsinki Declaration of 1975, as revised in 2008. This study was approved by the Central Mental Hospital (Dublin) Audit, Research and Ethics Committee having regard to the appropriate safeguards regarding patient protection and data security (approval reference REC/2607 2021/HK/021).

### Scale development

The excellence scale was constructed in four phases. First was a presentation at the International Association of Forensic Mental Health Services annual conference in New York in June 2017, followed by a discussion among experts. Second was an iterative drafting leading to the publication of an article including Table 1 describing the seven domains each with four levels.^1^ The third phase was an iterative consensus process drawing on international experts from a range of disciplines convened by H.G.K.,^33^ leading to the drafting of the excellence scale as a questionnaire. A fourth phase prioritised the views of translators and international collaborators to ensure that form and content were as universal as possible and independent of local factors.

### Survey

A survey was constructed using the Qualtrics software survey package and administered online (Qualtrics, Provo, Utah). The survey is self-rated, cross-sectional and mixed qualitative and quantitative in nature. It took approximately 20 min to complete. The survey questions are derived from the excellence framework (Table 1) and collect data from each participating service on each of the seven domains described. This also included information on levels of security, the number of in-patients and out-patients, the number of forensic psychiatrists, the number of prison in-reach teams, number of hospital teams and out-patient teams, links between hospitals and links between levels of security for patient pathways. Linkages for training and university research links were also counted. The survey did not collect any patient-specific information.

All items were scored 0 (absent) or 1 (present). Items in each domain were added and domain scores standardised by dividing the number of items in each domain to yield a score in the range 0 to 1.

The number of levels of therapeutic security co-located on one campus, with the addition of community aftercare services where present, was calculated counting one each for high, medium, low and community services, giving a range of 1 to 4.

The study involved collaboration with multiple forensic mental healthcare centres in Belgium, Denmark, Ireland, Italy, and Ontario, Canada. An identified academic forensic lead in each country assisted with survey distribution and provided a link to each of the participating forensic centres in each country. Each site received the same questions in the same format, and the methodology was standardised across each site and each country.

The survey was distributed to forensic mental health services only. Forensic leads (either clinical or academic) in each participating service were asked to complete the survey. A second data-checking round was conducted with the principal investigators for each jurisdiction.

### Statistical analysis

All data were analysed using IBM SPSS Statistics 28.0 for Windows. We tested the psychometric properties of the excellence scale to demonstrate factorial and internal consistency and conceptual coherence while also seeking real-world features of forensic psychiatry services (content validity) that might relate to excellence.

Factor analysis and Cronbach's alpha were used to demonstrate internal consistency and conceptual coherence, and a reliable change index (RCI) was calculated;^34,35^ we then carried out dimensional checks and statistical modelling using correlation, analysis of variance (ANOVA) and linear regression analysis. Only aggregated data are presented to preserve the anonymity of the respondents and participating services.

### Reliable change index and meaningful change

An RCI was calculated to allow individual services to evaluate differences and to evaluate change over time. This is a more useful parameter than a power calculation, given the absence of previous studies and because one of the purposes of the scale is to allow services to compare themselves against their own previous scores. If the RCI is less than 0.25 units for the total mean score, this suggests that the meaningful distinctions of basic (0–0.25), standard (0.26–0.5), progressive (0.51–0.75) and excellent (0.76–1.0) can be interpreted for the mean total score with reference to the RCI.

### Dimensional consistency

If the characteristics of each level are consistent across domains, then analysis of trend is expected to show a progressive fall in mean scores from level 1 to level 4.

### Statistical modelling of determinants of excellence scores

We examined whether independent characteristics of forensic services might influence the total excellence score and scores for domains of excellence. We first analysed correlations between independent variables, characteristics of the 48 services, then used ANOVA to check for significant differences between groups. In an exploratory modelling exercise using linear regression analysis, the total excellence score (excluding the ‘timescale’ domain) was used as the dependent variable and the predictor variables entered were those that differed significantly for total excellence score. A preliminary ‘enter’ method was used, then both backward and forward entry methods were applied to check for robustness. Because of a possibly circular relationship between some apparent determinants of the excellence scale and some domains of the scale, a *post hoc* regression analysis was carried out with each domain as the dependent variable.

## Results

### Sample and service characteristics

A total of 48 survey responses were obtained across the five participating jurisdictions (Table 2). The 48 secure forensic services that completed questionnaires represented full coverage of all forensic mental healthcare sites in Ireland, Denmark and Ontario, and approximately 46 and 64% in Italy and Belgium respectively. Lead investigators in each jurisdiction ensured that each completed response set was independent of all of the others and masked to all of the others; consequently, the sample consists of 48 independent sets of responses.

**Table 2:** Survey responses

Country	Responses, *n* (% of total)
Ireland	1 (100%)
Denmark	6 (100%)
Italy	23 (46%)
Belgium	9 (64%)
Ontario	9 (100%)
Total	48

As regards level of therapeutic security, units described themselves as high secure (*n* = 4, 8%), medium secure (*n* = 33, 69%) or medium and low secure combined (*n* = 11, 23%). High secure units served larger catchment area populations (high secure: mean 6.9 million, s.d. = 2.7 million; medium secure: mean 1.7 million, s.d. = 1.8 million; medium and low secure co-located: mean 2.4 million, s.d. = 2.2 million; ANOVA *F* = 8.3, d.f. = 2, *P* = 0.001, Bonferroni correction for high versus others *P* = 0.008). Medium secure units that were not co-located with high or low secure units had fewer beds (high secure: mean 176.5, s.d. = 75.3; medium secure: mean 41.6, s.d. = 45.3; medium and low secure co-located: mean 113.8, s.d. = 54.2; ANOVA *F* = 18.7, d.f. = 2, *P* < 0.001, Bonferroni correction medium versus others *P* < 0.001). Number of out-patients was higher for co-located medium and low secure services but this did not reach significance (high secure: mean 87.5, s.d. = 103.1; medium secure: mean 48.5, s.d. = 108.1; medium and low secure combined: mean 110.2, s.d. = 89.1; ANOVA *F* = 1.4, d.f. = 2, *P* = 0.249). The high standard deviations can be taken as evidence for wide variations within each category.

Of the 48 services, 20 (41.7%) had only one level of therapeutic security, 15 (31.3%) had two levels, 10 (20.8%) had three levels and 3 (6.3%) had four levels.

### Factor analysis

An initial exploratory factor analysis demonstrated that items in the ‘timescale’ domain had insufficient variation to allow computation. Further analysis yielded a solution in which the level 1 items for each remaining domain loaded weakly or in a way that was not consistent. A factor analysis omitting them produced a solution in which all remaining items loaded strongly onto the first component, accounting for 38% of the variance, and remaining components had no meaningful interpretation. A varimax rotation produced four factors but again all items loaded best onto the first factor and remaining factors had no meaningful interpretation.

Each domain score had skewness in the range −0.474 to +0.661 and kurtosis in the range −0.893 to −0.063 except for time, which had a kurtosis of +3.73. We therefore eliminated the ‘timescale’ items from calculations.

### Psychometric properties of the excellence scale

Psychometric properties of the excellence scale were calculated based on all 48 survey responses.

Internal consistency was demonstrated for each level summed across domains, from level 1 through to level 4, with level 1 the least internally consistent (Table 3). Each service can be allocated a score for each level that is reliable. The expected results would be that in general, for any individual unit there should be a decreasing trend from level 1 to level 4 (Table 4).

**Table 3:** Internal consistency for the four levels of excellence^a^

Level	Interpretation		Items^b^	Cronbach's alpha
Level 1	Basic	Individual expertise	1.1 to 7.1 (omit 4.1)	0.591
Level 2	Standard	Local multidisciplinary teams	1.2 to 7.2 (omit 4.2)	0.720
Level 3	Progressive	Integrated services	1.3 to 7.3 (omit 4.3)	0.668
Level 4	Excellent	Academically led and international networks	1.4 to 7.4 (omit 4.4)	0.783
Cumulative		All of the above	All the above	0.904

a. Domains: 1 Values and rights; 2 Clinical organisation; 3 Consistency; 4 Timescale; 5 Specialisation; 6 Routine outcome measures; 7 Research and development.

b. Each domain is made up of Items. Each item is scored in four levels. Item numbers refer to domain number and excellence level.

**Table 4:** Analysis of trend for excellence scores (levels 1 to 4) for the 48 forensic units

Level of excellence	Mean excellence score	s.d.	95% CI	
			Lower	Upper
Level 1	0.674	0.187	0.619	0.728
Level 2	0.542	0.201	0.484	0.601
Level 3	0.515	0.178	0.463	0.566
Level 4	0.407	0.200	0.348	0.465

### Analysis of trend

General linear modelling across levels (Table 4) yields multivariate tests all with *F* = 33.2 (d.f. = 3/45), *P* < 0.001 and η^2^ = 0.689. Tests of within-subjects effects all yield *F* = 54.2, *P* < 0.001, partial η^2^ = 0.536. Within-subjects contrasts show that linear contrast was significant (*F* = 90.3, d.f. = 1, *P* < 0.001, partial η^2^ = 0.658), quadratic contrast was not significant (*F* = 0.707, d.f. = 1, *P* = 0.405, partial η^2^ = 0.015) but cubic contrast was significant (*F* = 19.4, d.f. = 1, *P* < 0.001, partial η^2^ = 0.292). This supports both a significant linear trend and a pattern with two changes of direction, between levels 1 and 2 (basic to standard) and between levels 3 and 4 (progressive to excellent). Tests of between-subjects effects are also significant (*F* = 476.6, d.f. = 1, *P* < 0.001, partial η^2^ = 0.910).

Internal consistency was also demonstrated for each domain (values and rights, clinical organisation, consistency, timescale, specialisation, routine outcome measures, and research and development; Table 5). The ‘timescale’ domain (which was eliminated from later calculations) had a negative value for Cronbach's alpha. Cronbach's alpha was lower for each domain when calculated for all four levels when compared with levels 2, 3 and 4 only. Each service can therefore be allocated a score on each domain that is reliable. Scores for each domain work best omitting the level 1 scores because of interpretative and directional problems.

**Table 5:** Internal consistency and reliable change index (RCI) for seven primary domains

Domain	Items included^a^	Mean excellence score	s.d.	Cronbach's alpha	RCI
1 Values and rights	1.1 to 1.4	0.58	0.26	0.899	0.23
2 Clinical organisation	2.1 to 2.4	0.59	0.17	0.580	0.30
3 Consistency	3.1 to 3.4	0.67	0.21	0.675	0.33
4 Timescale	4.1 to 4.4	0.76	0.09	−0.059	-
5 Specialism	5.1 to 5.4	0.49	0.19	0.660	0.31
6 Outcome measures	6.1 to 6.4	0.44	0.21	0.596	0.37
7 Research and development	7.1 to 7.4	0.43	0.28	0.918	0.28
Mean total domain score	1.1 to 7.4	0.56	0.15	0.896	0.13
Mean total domain score, excluding ‘timescale’	All above except 4.1 to 4.4	0.53	0.17	0.904	0.15

a. Item numbers refer to domain number and excellence level (levels 1–4).

### Reliable change index

The reliable change criterion (reliable change index, RCI) is in the range 0.23 to 0.38 for all domains and is 0.15 for the total mean score, suggesting that the meaningful distinctions of basic (0–0.25), standard (0.26–0.5), progressive (0.51–0.75) and excellent (0.76–1.0) can be interpreted for the mean total score with reference to the RCI.

### Determinants of excellence

**Fig. 1:**
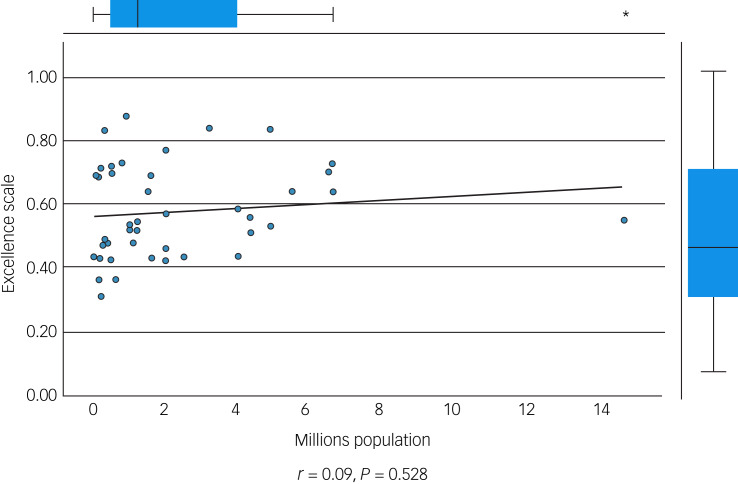
Population served and excellence score.

**Fig. 2:**
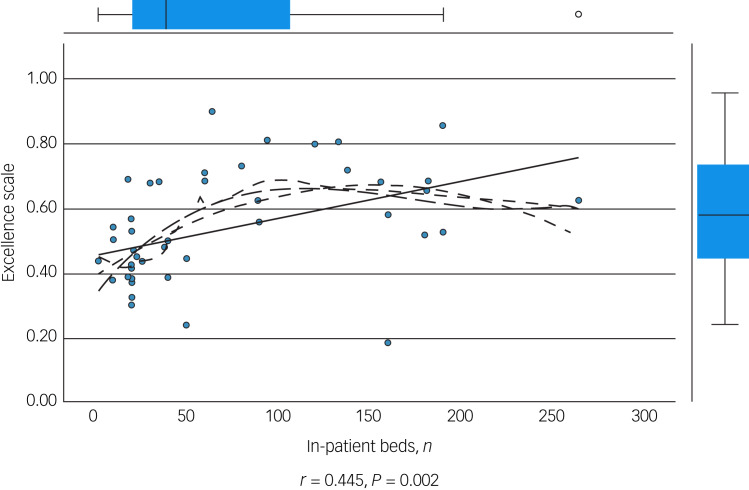
In-patient bed numbers and excellence score.

**Fig. 3:**
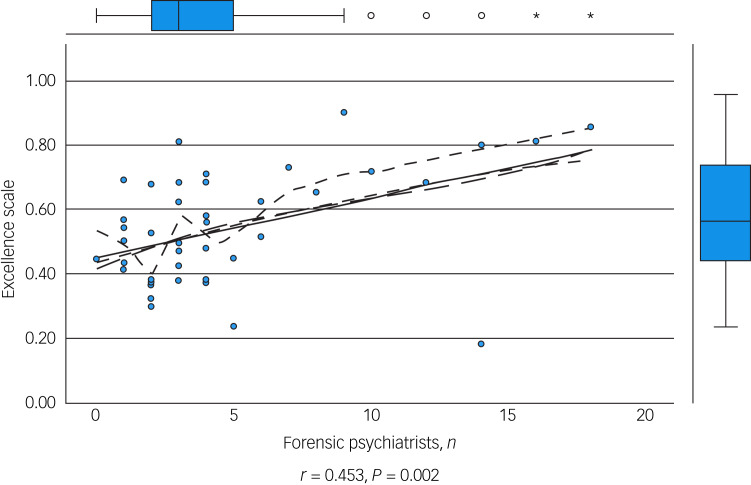
Number of forensic psychiatrists and excellence score.

**Fig. 4:**
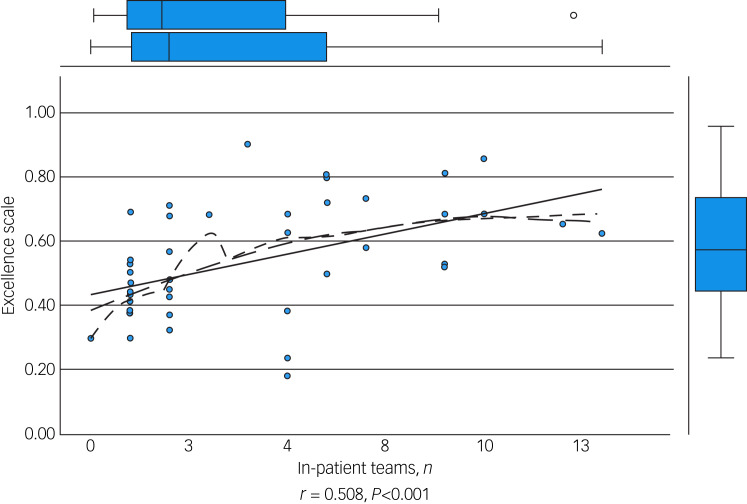
Number of in-patient teams and excellence score.

**Fig. 5:**
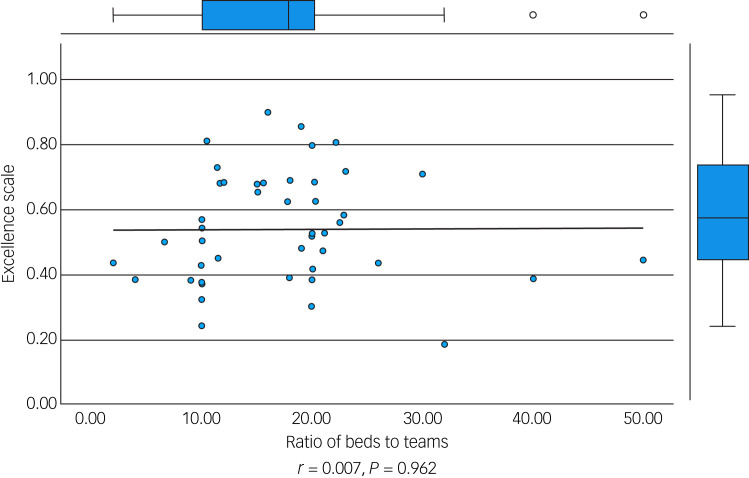
Ratio of beds to teams and excellence score.

**Fig. 6:**
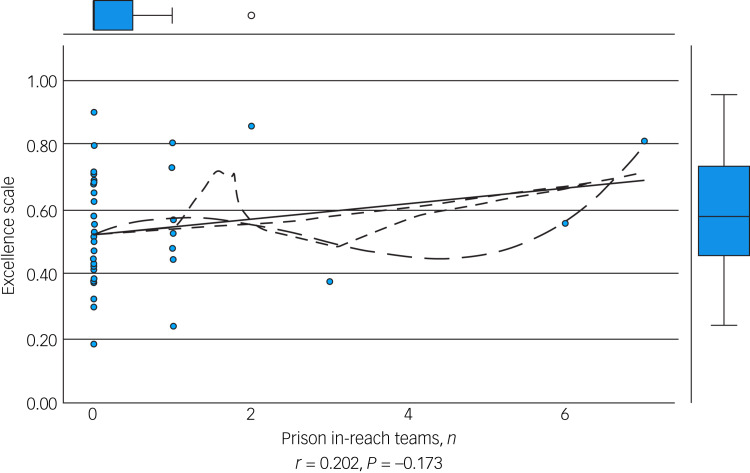
Number of prison in-reach teams and excellence score.

**Fig. 7:**
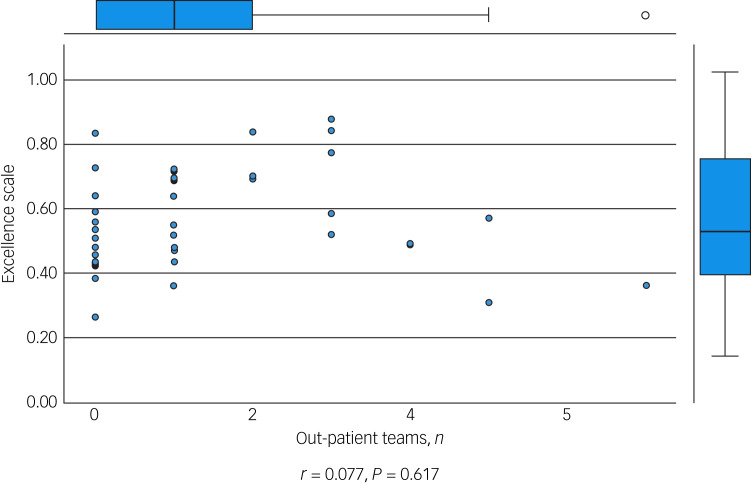
Number of out-patient teams and excellence score.

We performed an initial exploratory correlation analysis (Table 6) and ANOVA prior to regression analysis. The mean total excellence score (excluding the ‘timescale’ domain) did not correlate with population served (Fig. 1) but did correlate significantly with: levels of therapeutic security, number of secure beds (Fig. 2), number of forensic psychiatrists (Fig. 3), number of in-patient teams (Fig. 4), having linked patient pathways, links with training and development, links with university research and joint university appointments. It did not correlate with ratio of in-patient beds to teams (Fig. 5), population served (Fig. 1), number of out-patients, number of prison in-reach teams (Fig. 6) and number of out-patient teams (Fig. 7). ANOVA confirmed significantly higher mean total excellence scores for linked patient pathways, training and development links, university research links and joint university appointments, with all differences of means exceeding the RCI (Table 7).

**Table 6:** Mean total excellence score correlated with independent variables^a^

Variable	Number of responses	Correlation coefficient *r*	95% CI of *r*		*P*
			Lower	Upper	
Population served (*n*)	43	0.099	−0.208	0.388	0.528
Level of security (1–4)	48	0.478	0.224	0.671	<0.001
Secure beds (*n*)	48	0.445	0.184	0.647	0.002
Out-patients (*n*)	48	0.227	−0.015	0.522	0.063
Forensic psychiatrists (*n*)	46	0.453	0.187	0.657	0.002
In-patient teams (*n*)	47	0.508	0.258	0.694	<0.001
Ratio of secure beds to in-patient teams	46	0.007	−0.284	0.297	0.962
Prison in-reach teams (*n*)	47	0.202	−0.090	0.463	0.173
Linked in-patient pathways (yes/no)	47	0.409	0.274	0.699	0.004
Links for training and development (yes/no)	47	0.397	0.124	0.614	0.006
University research links (yes/no)	48	0.460	0.203	0.659	<0.001
Joint university appointments (yes/no)	48	0.518	0.274	0.699	<0.001

a. The mean total excellence score excluded the ‘timescale’ domain.

**Table 7:** Independent variables and mean total excellence score^a^

Variable	Not present			Present			ANOVA	
	*n*	Mean	s.d.	*n*	Mean	s.d.	*F*	*P*
Linked patient pathways	9	0.39	0.009	38	0.56	0.17	9.03	0.004
Training and development links	19	0.45	0.14	28	0.59	0.17	8.42	0.006
University research links	22	0.45	0.14	26	0.61	0.16	12.36	<0.001
Joint university appointments	30	0.47	0.14	18	0.65	0.16	16.88	<0.001

a. The mean total excellence score excluded the ‘timescale’ domain.

**Fig. 8:**
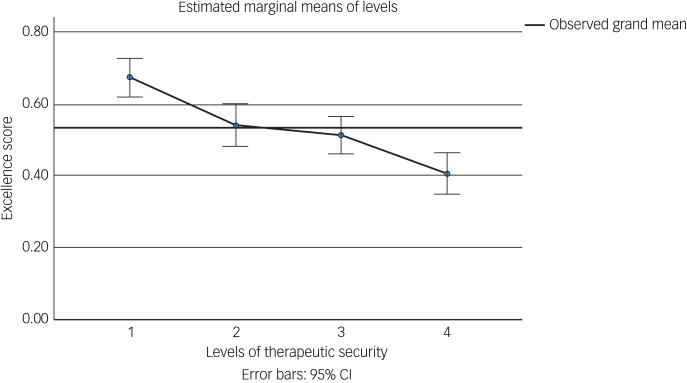
Number of levels of therapeutic security and excellence. Number of levels of therapeutic security includes community services.

The number of levels of therapeutic security (1 to 4) was also associated with an incrementally higher score for excellence (Fig. 8) (Table 8). Units with four levels of therapeutic security served larger catchment area populations. Units with more levels of therapeutic security had more secure beds and more in-patient teams, although this was accounted for by a fairly constant ratio of in-patient beds to teams (17.2 beds per team overall, *n* = 46, 95% CI 14.6–19.9). Similarly, units with more levels of therapeutic security had more out-patients under supervision and more out-patient teams, although again with a ratio of out-patients to teams that was fairly constant (range 46.7–113.8). The number of forensic psychiatrists for units with more levels of security was higher. Units with higher numbers of levels of therapeutic security were also more likely to have linked patient pathways, training and development links, university research links and joint university appointments (Table 8).

**Table 8:** Independent variable ‘number of levels of therapeutic security’ and mean total excellence score^a^

	Levels of therapeutic security, including community													
	One level			Two levels			Three levels			Four levels			ANOVA d.f. = 3	
	*n*	Mean	s.d.	*n*	Mean	s.d.	*n*	Mean	s.d.	*n*	Mean	s.d.	F	*P*
Mean total excellence score	20	0.44	0.11	15	0.58	0.18	10	0.61	0.18	3	0.69	0.15	5.02	0.004
Population served (millions)	18	2.88	2.19	13	0.70	0.43	9	2.11	2.13	3	6.80	7.14	5.8	0.002
Secure hospital beds	20	53.1	74.0	15	50.5	43.9	10	115.8	56.7	3	118.0	54.1	3.5	0.024
In-patient teams	20	3.2	3.3	14	2.6	2.6	10	6.3	3.2	3	8.3	1.2	5.4	0.003
Ratio of beds to in-patient teams	20	15.1	7.9	14	18.4	8.6	10	21.0	11.1	3	13.9	5.3	1.2	0.307
Out-patient numbers	20	0	0	14	110.9	142.9	9	116.3	92.2	3	135.0	73.7	6.2	0.001
Out-patient teams	20	0.15	0.37	13	2.1	2.1	10	2.6	1.4	2	1.5	0.7	9.7	<0.001
Ratio of out-patients to out-patient teams	3	0	0	11	46.7	44.7	9	53.3	43.1	2	113.8	121.9	2.2	0.115
Number of forensic psychiatrists	20	2.9	2.8	15	3.8	3.2	10	7.7	5.6	3	9.7	5.5	5.5	0.003
	***n***	**Positive**^b^	**%**	***n***	**Positive**	**%**	***n***	**Positive**	**%**	***n***	**Positive**	**%**	**χ^2^**	***P***
Linked patient pathways	20	12	60	14	13	92.9	10	10	100	3	3	100	9.9	0.019
Training and development links	20	10	50	14	7	50	10	8	80	3	3	100	5.1	0.017
University research links	20	7	35	15	11	73.3	10	5	50	3	3	100	7.8	0.051
Joint university appointments	20	2	20	15	8	53.3	10	5	50	3	3	100	13.7	0.003

a. The mean total excellence score excluded the ‘timescale’ domain.

b. ‘Positive' indicates that the item concerned (linked patient pathways, training and development links, university research links, joint university appointments) was present.

### Statistical modelling of determinants of excellence scale

The total excellence score was used as the dependent variable in linear regression analysis and independent variables were selected based on the earlier analyses of correlation and differences: namely, in-patient bed numbers, out-patient numbers, number of security levels, number of forensic psychiatrists, number of in-patient teams, linked patient pathways and joint university appointments. A preliminary ‘enter’ method generated a model with adjusted *R*^2^ = 0.889, *R*^2^ change 0.907, *F* change 51.4, d.f. = 7,37, *P* < 0.001 and ANOVA *F* = 51.4, d.f. = 7, *P* < 0.001. Only the beta coefficient for linked patient pathways reached significance (*t* = 3.98, *P* < 0.001, β = 0.254, 95% CI 0.126–0.388). Using both backward and forward entry methods to check for robustness, the forward entry method resolved in four iterations (adjusted *R*^2^ = 0.893, ANOVA *F* = 92.9, d.f. = 4, *P* < 0.001) with four predictor variables: number of security levels (*t* = 4.98, *P* < 0.0001), linked patient pathways (*t* = 4.07, *P* < 0.001), number of in-patient teams (t = 2.73, *P* = 0.009) and joint university appointments (*t* = 2.07, *P* = 0.045). Backward entry produced the same result, indicating a robust model.

### *Post hoc* analysis

Owing to potential circularity, a regression model was examined for each of the domains. Although each of these resolved in two or three iterations, the pattern was consistent. Number of levels of security predicted higher scores for values and rights, consistency and routine outcome measures; linked patient pathways predicted values and rights, clinical organisation, consistency, specialisation and research and development; joint university appointments predicted clinical organisation and research and development; number of in-patient beds predicted clinical organisation; and number of in-patient teams predicted specialisation and routine outcome measures.

## Discussion

The excellence scale was constructed to elicit scores on domains of excellence and generated measures with acceptable factorial coherence, internal consistency and reliable change criteria in this large international sample. Factor analysis indicated a single scale with high internal consistency and low reliable change index.

An initial exploratory factor analysis demonstrated that the ‘timescale’ items had insufficient variation. A factor analysis eliminating timescale items produced a solution in which all remaining items loaded strongly onto the first factor. Levels had good internal consistency except ‘basic’ and for each domain except ‘timescale’. Accordingly a single ‘excellence’ score was calculated and correlated with independent characteristics of forensic services, such as number of levels of therapeutic security, number of secure beds, number of forensic psychiatrists, linked patient pathways, and links with training and development, with university research and with joint university appointments. Regression analysis found that a model including number of security levels, linked patient pathways, number of in-patient teams and joint university appointments predicted total excellence score.

Internal consistency was demonstrated for each level of the excellence scale, from level 1 through to level 4. Each service can be allocated a score for each level that is reliable. Internal consistency was also demonstrated for each domain. Each service can therefore also be allocated a score on each domain that is reliable. The reliable change index (RCI) allows an individual service to compare itself against its own score in previous years. The division of the mean excellence score into broad levels (basic 0–0.25, standard 0.25–0.5, progressive 0.51–0.75 and excellent 0.75+) provides an index of meaningful change.

Larger units generally scored better, although ‘large’ never exceeded 250 secure beds. Those with community aftercare services, multiple levels of therapeutic security on one campus and integrated patient pathways also scored higher. Shared university appointments predicted higher scores, as did research links and training links. This is in keeping with evidence in many areas of medicine that acquiring and maintaining clinical expertise at the highest level requires a high clinical throughput and that hard outcomes are better in such services.^5,36–43^

It has not previously been shown that the number of forensic psychiatrists and number of in-patient teams in a service can be related to excellence. This suggests that critical mass has a beneficial effect on breadth and depth of skills, experience and expertise – size matters, smaller is not better, although it is again noted that no service in this survey had more than 250 beds, in keeping with other modern surveys.^11^

Having several security levels within a forensic mental health service is an expected feature of effective forensic psychiatry services,^44–48^ as are linked patient pathways in all health services,^49^ in forensic services ^50^ and in comprehensive mental health services that include forensic services and forensic pathways.^51–54^ It may also be that larger services with well-defined models of care^4^ inherently require or generate better governance structures for consistency and quality.

The benefit of joint university appointments has not previously been shown for forensic mental health services, although it has been demonstrated for many other specialist medical health services^28,55,56^ and is inherent in the OECD recognition of the need for specialist funding for university-affiliated hospitals in which teaching, training, research and development are almost impossible to separate.^26^

Our excellence scale has demonstrated that forensic mental health services can benchmark their current practices by comparison with an international basket of averages^7^ and even more meaningfully by continuous self-evaluation.

### Limitations

This study relied on self-assessment by the participating services for the excellence domains, although we were able to cross-check the independent service characteristics through consultation with the principal investigators in each jurisdiction. Reliability and validity could be improved with peer review and with objective measures such as quality and quantity of research output. In this paper we have not been able to test the excellence scale and its domains against any hard outcomes for patients.^4,27^ Assessing and tracking changes in a measure of excellence ultimately requires some independent validating evidence that it caused a benefit for patients. Because this study was designed to include only secure forensic services, there was insufficient variation in scores for the timescale domain. Including acute low secure or ‘locked’ psychiatric intensive care units or general adult admission units^44,53^ in a future study may correct this.

### Future directions

For the future, studies of other objective measures of better outcomes for patients, including shorter lengths of stay, reduced recidivism and readmission, and improved physical and mental health and quality of life, would be the best criteria for excellence. The growth of multi-centre and international networks for research and development would drive progress from routine outcome measurement towards developing consistent ‘treatment as usual’. Excellence in specialist education and training in such services should lead to better outcomes. These objective outcomes would be signs that measuring excellence and driving improvement in excellence is worthwhile.

Other applications of this tool may include comparative evaluation of models of care, clinical practice and service choice,^4,24,57^ commissioning by central funding and policy authorities,^26^ and regulatory and inspection functions. It can be used to ask ‘Where are we?’ and to learn from international experience.

### Implications for services

It is not enough that excellence should be an aspiration without a definition, practical processes or measurable goals. This conceptual framework for excellence generated a measure of excellence that had good psychometric properties in this large international sample. Larger services organised according to stratified therapeutic security and with strong university and research links scored higher on this measure. We believe that using these self-ratings to set goals for progression to higher levels in each domain would form the basis for an improvement cycle. Linking these self-ratings to objective outcomes such as scientific publication numbers and quality, and outcomes of research and development, teaching and training would represent the next stage in validation. A further step in validation would be to link these domains of excellence to improving service levels at population level, to productivity and unit cost, mortality, length of stay, measurable health gains and safety measures. Does increased volume allow reduced unit costs with the same quality outcomes, or increased quality at the same unit cost? Pedersen^57^ suggests they do not, because the logic of economics is not the same as the logic of professional practice, while noting the importance of careful modelling in non-linear systems.

In many jurisdictions, forensic mental health services are the only hospital and community services for the most severely ill psychiatric patients. These settings are the only ones capable of delivering the most intensive and complex treatments sustained over the periods of time necessary for change under closely monitored and evaluated conditions.^4,27^ We believe that forensic mental health services can drive excellence in outcomes for patients by defining ‘treatment as usual’, pursuing research-informed outcome measurement and management and continuously improving treatment as usual, translating research into specialist education, training and practice while observing real-world outcomes. Early indications are that larger forensic hospitals with joint university appointments are more likely to have excellent values, clinical organisation, consistency, specialisation, routine outcome measurement and research activity. It would be useful also to quantify the effect that university-linked forensic psychiatry teams have on their neighbours within the same hospital on neighbouring services.^29,30,49,58^ There is evidence that centres of excellence in oncology generate such benefits. The ambition in forensic mental health services should be to emulate the advances made by such networks.

## Data Availability

The data that support the findings of this study are available on request from the corresponding author. The data are not publicly available.
